# Emerging roles of long noncoding and circular RNAs in pancreatic ductal adenocarcinoma

**DOI:** 10.3389/fphys.2022.1025923

**Published:** 2022-11-14

**Authors:** Hiromichi Sato, Tomoaki Hara, Shotaro Tatekawa, Kazuki Sasaki, Shogo Kobayashi, Toru Kitagawa, Yuichiro Doki, Hidetoshi Eguchi, Kazuhiko Ogawa, Shizuka Uchida, Hideshi Ishii

**Affiliations:** ^1^ Department of Gastrointestinal Surgery, Osaka University Graduate School of Medicine, Suita, Japan; ^2^ Department of Medical Data Science, Center of Medical Innovation and Translational Research, Osaka University Graduate School of Medicine, Suita, Japan; ^3^ Department of Radiation Oncology, Osaka University Graduate School of Medicine, Suita, Japan; ^4^ Kyowa-kai Medical Corporation, Osaka, Japan; ^5^ Aalborg University, Aalborg, Denmark

**Keywords:** ncRNA, pancreas, cancer, inflammation, therapy

## Abstract

An international project on the human genome revealed that various RNAs (e.g., messenger RNAs, microRNAs, and long noncoding RNAs [lncRNAs] and their subclass circular RNA [circRNA)) are involved in the pathogenesis of different human diseases, including cancer. Recent studies have highlighted the critical roles of lncRNAs and circRNA in pancreatic ductal adenocarcinoma (PDAC), especially in the epithelial–mesenchymal transition, a phenomenon regulating cancer metastasis. Growing research in this field has indicated that the tertiary structure of lncRNAs supposedly regulates biological function *via* RNA–RNA or RNA–protein associations, aiding early diagnosis and therapy selection for various diseases, including cancer. Here we describe the emerging roles of ncRNAs in PDAC and highlight how these ncRNAs can be used to detect and control this intractable cancer.

## 1 Introduction

**FIGURE 1:**
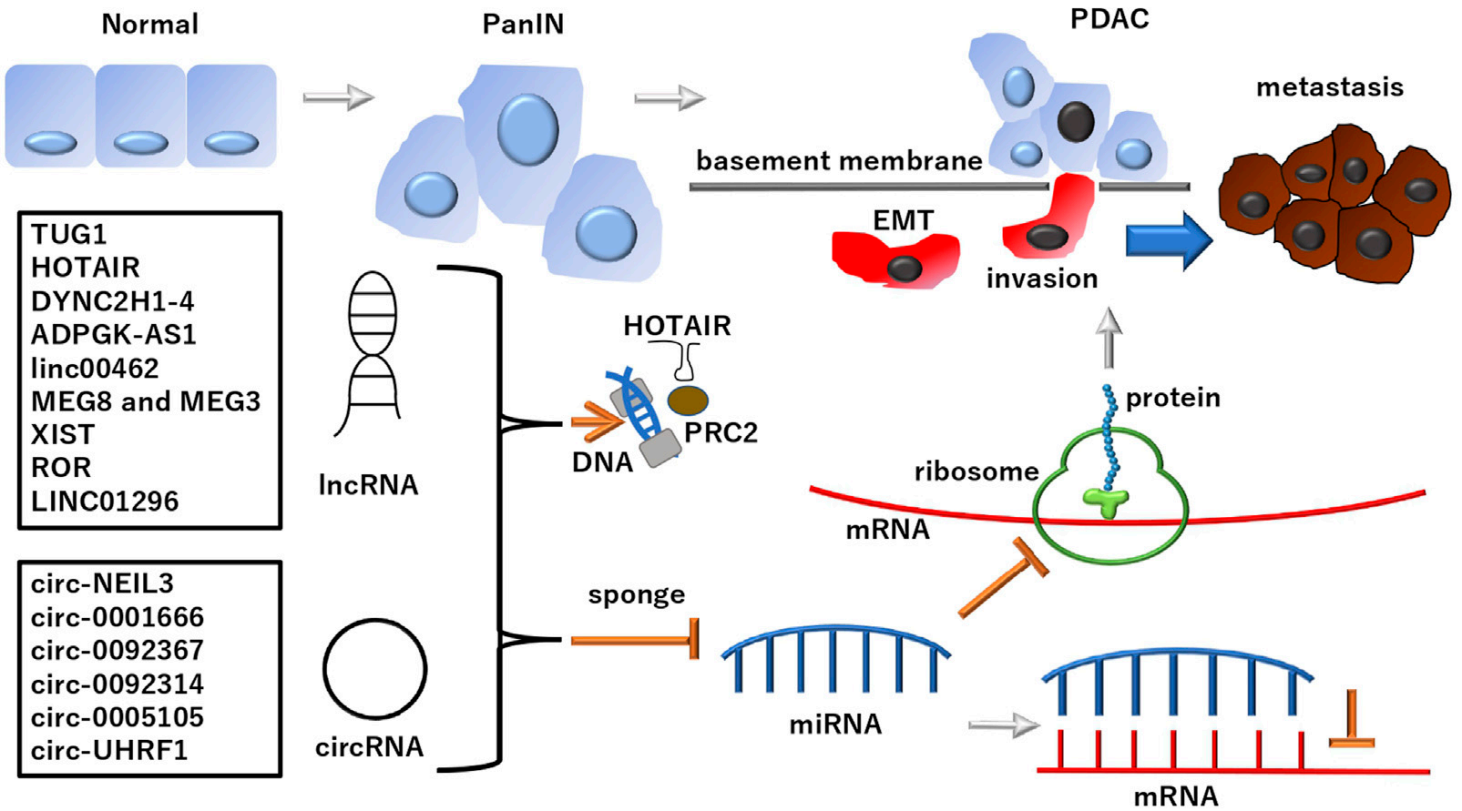
lncRNAs and subclass circRNAs in PDAC PDAC developed from normal ducts in the pancreas and its altered precancer lesion, pancreatic intraepithelial neoplasia (PanIN) (Suryavanshi et al., 2017; Ma et al., 2021). lncRNAs and subclass circRNAs can suppress the miRNA-dependent inhibition of mRNA transcripts and their translated protein products involved in the development of PDAC. Notably, lncRNAs and circRNAs play roles in EMT regulation, which is involved in PDAC metastasis: A phenomenon causing fatal outcomes in patients. Although lncRNAs and circRNAs deploy various mechanisms and functions, as mentioned in the text, the schematic of a typical pathway is depicted in this figure. The multi-faced function of ncRNAs includes the involvement in epigenetic regulation for PRC2 by HOTAIR. See each abbreviation in-text. lncRNA, long noncoding RNA; circRNA, circular RNA; PRC2, polycomb repressive complex 2; PDAC, pancreatic ductal adenocarcinoma.

Cancer, a genetic disease, involves multiple mutations in cell growth-promoting and death-inhibiting oncogenes and growth-restricting tumor suppressor genes. These mutations arise from various genetic alterations, including those in both coding and noncoding regions of chromosomes (Nowell, 1993; Hanahan and Weinberg, 2000; Hanahan and Weinberg, 2011). Positional cloning approaches for exploring oncogenes and tumor suppressor genes have enabled researchers to identify multiple transcripts exhibiting aberrant structures and expression levels (Hanahan and Weinberg, 2000; Hanahan and Weinberg, 2011). MicroRNAs (miRNAs), a type of short noncoding RNA, were first discovered in a study regarding hematopoietic malignancies (Calin et al., 2002; Calin et al., 2005). Only a portion of the transcripts in human cells are associated with protein-coding genes. Long noncoding RNAs (lncRNAs) are at least four times more transcribed than protein-coding transcripts (Kapranov et al., 2007). The human genome project identified various transcripts, including lncRNAs. The findings of this project identified that large-scale cDNA sequencing projects can reveal transcriptional complexities (Carninci et al., 2005). Generally, lncRNAs are defined as transcripts of >200 nucleotides that have been considered, although it was discussed, not translated into protein (Kung et al., 2013). According to multiomics analyses, tens of thousands of lncRNAs are potentially associated with various diseases, providing further evidence in their involvement and contribution in neurological disorders and cancer (Ma et al., 2019). In this opinion article, we focused on the recent advances in understanding the role of lncRNAs and their corresponding nucleotides in a typical refractory disease in gastrointestinal organs, pancreatic ductal adenocarcinoma (PDAC), whose epidemiology has been described in the Discussion section (Figure 1).

## 2 LncRNAs in PDAC

Although ncRNA are generally defined as nonprotein-coding, notably, previous ribosome profiling studies suggest that 40%–90% of the annotated lncRNAs undergo translation (Ingolia et al., 2011; Guttman et al., 2013; Ji et al., 2015), indicating diverse functions of ncRNAs. A study involving mouse embryonic stem cells aimed to reveal the complexity and dynamics of mammalian proteomes and defined a class of short, polycistronic ribosome-associated coding RNAs encoding small proteins known as micropeptides (Ingolia et al., 2011). A study involving lncRNAs, 5'UTRs, and pseudogenes indicated that lncRNAs may be translated, with some likely to express functional peptides or proteins (Ji et al., 2015). However, the peptides generated from lncRNAs translation are unstable and lack biological function (Ji et al., 2015). Nevertheless, there is a lack of consensus regarding the exact method for assessing whether RNAs express functional peptides or proteins (Guttman et al., 2013).

Although several lncRNAs may be involved in generating small functional proteins or peptides, sequence complementarity to other nucleotides probably helps regulate target stability or function (Yang et al., 2022). lncRNAs act as competing endogenous RNAs (ceRNAs) to sequester target miRNAs, bind nearby target genes, and directly control epithelial–mesenchymal transition (EMT)-related proteins (Yang et al., 2022); EMT characterizes the metastasis and invasion of cancer cells (Pastushenko and Blanpain, 2019). It is not an on-or-off binary process but occurs in distinct cellular states, involving multiple continuous dynamic transcriptional and translational mechanisms (Pastushenko and Blanpain, 2019). EMT and its reverse phenotype (mesenchymal–epithelial transition) are transcriptionally regulated at the epigenetic level by mRNAs, miRNAs, and lncRNAs (Brabletz et al., 2021). The transcription factor cascade involved includes snail family transcriptional repressor 1 (*SNAI1*), *SNAI2*/*SLUG*, TWIST family basic helix-loop-helix transcription factor 1, zinc finger E-box-binding homeobox 1 (*ZEB1*), and *ZEB2* (Brabletz et al., 2021). Therapeutically targeting EMT interferes with tumor progression (Brabletz et al., 2021). By combining single-cell RNA and protein analytics to investigate the role of stromal cancer-associated fibroblasts in PDAC, considerable single-cell population shift toward EMT and proliferative phenotypes linked with mitogen-activated protein kinase and signal transducer and activator of transcription 3 have been identified (Ligorio et al., 2019; Brabletz et al., 2021). These observations highlight the influence of the stroma in shaping tumor architecture in PDAC, which is closely associated with the clinical observation that PDAC invasion and metastasis occur during the early stages of the disease (Ligorio et al., 2019). Generally, PDAC already metastasizes to multiple organs by its diagnosis (Chen et al., 2021). Hence, to further understand the disease mechanism of PDAC, the investigation of lncRNAs is warranted.

### 2.1 lncRNAs function as CeRNAs in PDAC

A recent study indicated that 50% lncRNAs in PDAC supposedly function as ceRNAs, which are any RNAs that bind other RNAs, such as miRNA sponges, thereby regulating other transcripts by competing for shared target sequences in miRNAs (Salmena et al., 2011; Yang et al., 2022). The ceRNA hypothesis proposes that RNAs form a large-scale regulatory network across the transcriptome, extensively communicating functional genetic information in the whole genome, and play important roles in various disease pathologies, including cancer (Salmena et al., 2011). Considering that a finite number of lncRNAs exist, the ceRNA hypothesis supports the exchange of information between molecules for communication within a closed space (Salmena et al., 2011). Of the remaining lncRNAs in PDAC, 9% are affected by the local regulators of their nearby genes (Yang et al., 2022), suggesting that transcriptomal changes in lncRNAs are also involved in the transcriptional regulation of other genes. Hence, lncRNAs can be used to assess the epigenetic regulation of local gene expressions. We discuss the ceRNA related function of lncRNAs and circRNAs.

### 2.2 lncRNAs involved in EMT in PDAC

A recent study indicated that 34% lncRNAs in PDAC are involved in regulating EMT or transforming growth factor beta 1 (TGFB)-related mechanisms (Yang et al., 2022), although lncRNAs are not mutually exclusive to the ceRNA functional group and overlap with each other.

#### 2.2.1 Taurine-upregulated gene 1 (TUG1)

TUG1, an lncRNA overexpressed in PDAC, was initially identified as an upregulated transcript by taurine; its abnormal expression has been reported in numerous cancers (Qin and Zhao, 2017; Zhao et al., 2017). TUG1 functions as an oncogenic lncRNA promoting tumor progression by functioning as an endogenous “sponge” and competing for *miR-382* binding to the target enhancer of zeste 2 polycomb repressive complex 2 subunit (*EZH2*), and regulates EMT-related gene expression *via* epigenetic control (Qin and Zhao, 2017; Zhao et al., 2017).

#### 2.2.2 HOX transcript antisense RNA (HOTAIR)

*HOTAIR* is located within the homeobox C (*HOXC*) gene cluster on chromosome 12 and is coexpressed alongside the *HOXC* genes (Ji et al., 2018). *HOTAIR* can bind lysine-specific demethylase 1 and polycomb repressive complex 2, serving as a scaffold for the assembly of these regulators at the *HOXD* gene cluster, thereby promoting the epigenetic repression of *HOXD* genes (Ji et al., 2018). In addition, *HOTAIR* interacts with *miR-17-5p*, which is a tumor promoter or suppressor depending on the cellular context. The interaction between HOTAIR and miR-17-5p includes polycomb repressive complex 2 (PRC2)-mediated chromatin regulation (Cloonan et al., 2008; Ji et al., 2018). The first reported and most well-studied oncomiR is the human *miR-17-92* polycistron, which is a cluster of seven miRNAs derived from the c-myc-regulated *c13orf2*5 locus at chromosome 13q31.3 (He et al., 2005). *miR-17-5p* regulates the G1/S phase cell cycle transition (Cloonan et al., 2008). The high expression in multiple tumors, including PDAC (Tang et al., 2021; Yang et al., 2022), makes *HOTAIR* a promising therapeutic target. Indeed, silencing lncRNA *HOTAIR* inhibits EMT and PDAC progression through the Wnt/β-catenin signaling pathway, providing a novel therapy for PDAC (Tang et al., 2021).

#### 2.2.3 DYNC2H1-4

PDAC is characterized by the overexpression of lncRNA DYNC2H1-4 (human chromosome 11q22), which subsequently promotes EMT and the subpopulation of cancer stem-like cell phenotypes by acting as a miR-145 sponge in pancreatic cancer cells, potentially associated with malignant behavior and chemoresistance (Gao et al., 2017).

#### 2.2.4 ADP-dependent glucokinase antisense RNA 1 (ADPGK-AS1)

lncRNA *ADPGK-AS1* (human chromosome 15q24.1) overexpression promotes PDAC progression *via* the ceRNA mechanism involving *miR-205-5p* by activating ZEB1-mediated EMT (Song et al., 2018), suggesting a link between cancer glycolysis control and EMT induction.

#### 2.2.5 linc00462

lncRNA *linc00462* (human chromosome 13q14.2) promotes the invasiveness of PDAC *via* the *miR-665*/TGFBR1-TGFBR2/SMAD2/3 pathway (Zhou et al., 2018), suggesting that a ceRNA mechanism links *linc00462* with the TGFB pathway.

#### 2.2.6 *MEG8* and *MEG3*

lncRNA *MEG8* (human chromosome 14q32) overexpression in lung cancer and PDAC suppresses *miR-34a* and *miR-203* expression, thereby upregulating the transcription factors *SNAI1* and *SNAI2*, consequently repressing cadherin 1/E-cadherin expression (Terashima et al., 2018). *MEG8* associates with EZH2 to recruit it to the regulatory regions of the two miRNAs, eliciting histone H3 methylation and transcriptional repression; the ceRNA mechanism in this case involves lncRNA–protein binding, thereby reducing the proportion of miRNAs (Terashima et al., 2018). The study indicated that endogenous *MEG8* lncRNA was indispensable for TGFB-induced EMT (Terashima et al., 2018), proposing it as a therapeutic target. MEG8 shares the delta-like homolog 1 gene (*DLK1*) and type III iodothyronine deiodinase gene (*DIO3*) locus with MEG3 in EMT regulation (Terashima et al., 2018). The imprint regulation of the *DLK1-DIO3* locus at 14q32.1–32.31 is biologically important for fetal development, wherein imprinting errors can cause disorders, such as cancer. Emerging evidence implicates this locus in both fetal organ and tumor development (Enterina et al., 2017). *MEG3* (human chromosome 14q32.2) is a maternally expressed imprinted gene (He et al., 2017). Reportedly, MEG3 can be affected by interaction of the activities of tumor protein 53 (TP53), mouse double minute 2 (human homolog), growth differentiation factor 15, retinoblastoma 1, and other key cell cycle regulators, suggesting that it can be used for cancer diagnosis and prognosis (He et al., 2017), despite the underlying ceRNA mechanism not being understood completely.

#### 2.2.7 X-inactive specific transcript (XIST)

lncRNA *XIST* (human chromosome Xq13.2) overexpression in PDAC promotes cancer cell migration, invasion, and EMT *via* a typical ceRNA mechanism involving the sponging of *miR-429* to modulate *ZEB1* expression (Shen et al., 2019).

#### 2.2.8 Regulator of reprogramming

lncRNA *ROR* (human chromosome 18q21.31) overexpression in PDAC promotes EMT *via* the ZEB1 pathway (Zhan et al., 2016). ROR promotes the proliferation, migration, and invasion of PDAC cells *via* the Salvador–Warts–Hippo/yes-associated protein (YAP) pathway (Chen et al., 2020), a mechanism different from that of ceRNA. YAP potentially mediates EMT in PDAC and can be an underlying target of *ROR*, which is supposedly an PDAC biomarker (Chen et al., 2020).

#### 2.2.9 LINC01296

lncRNA *LINC01296* (human chromosome 14q11.2) overexpression in PDAC promotes cell metastatic properties by influencing EMT, indicating a poor PDAC prognosis, whereas its silencing elicits apoptosis by impacting the B-cell chronic lymphocytic leukemia/lymphoma 2/caspase-3 pathway (Yuan et al., 2019), thereby suggesting the suitability of this lncRNA as a diagnostic and therapeutic target of PDAC.

#### 2.2.10 MALAT1

The lncRNA, metastasis*-*associated lung adenocarcinoma transcript 1 (*MALAT1*; human chromosome 11q13.1) is conserved evolutionary (Johnsson et al., 2014). *MALAT1* is transcribed from a single exon into a 7-kb long RNA molecule as a precursor transcript. *MALAT1* is derived by RNase *p* cleavage of a tRNA-like small ncRNA (mascRNA) from its 3' end (McCown et al., 2019). The resultant mature transcript lacks a canonical poly(A) tail but is stabilized by a 3' triple helical structure (McCown et al., 2019). Whereas the processed *MALAT1* is predominantly retained in the nuclear speckles, mascRNA is transferred into the cytoplasm (Johnsson et al., 2014; McCown et al., 2019). Although the function of *MALAT1* in “miRNA sponge” was noted and reviewed (Plotnikova et al., 2019), the involvement of PDAC remains to be investigated fully.

## 3 Circular RNAs in PDAC

During normal splicing, introns are removed from premRNA to form mRNA. However, back splicing can induce one or more exons to form a single ring, thereby generating circRNAs (Barrett and Salzman, 2016; Patop et al., 2019), a subclass of noncoding RNAs (Qu et al., 2015). Certain circRNAs interact with miRNAs, whereas some others are translated. Furthermore, circRNAs reportedly regulate immune responses (Patop et al., 2019). Recent studies have proposed that circRNAs alongside lncRNAs participate in EMT in PDAC, affecting the migration and invasion of tumor cells by playing important roles in epigenetic processes, transcription, and post-transcriptional regulation (Yang et al., 2022). However, the mechanisms underlying their intelligent structures and whether they can correctly and efficiently act as miRNA sponges remain unclarified (Qu et al., 2015; Barrett and Salzman, 2016; Patop et al., 2019). A significant fraction (>90%) of circRNAs supposedly functions as ceRNAs against target miRNAs through RNA–RNA complementarity (Yang et al., 2022).

### 3.1 circ-NEIL3

*circ-NEIL3* (human chromosome 4 [chr4]:178274462-178,281,831) overexpression in PDAC facilitates cancer proliferation and metastasis through *circ-NEIL3*/*miR-432-5p*/adenosine deaminases acting on the RNA 1 (ADAR1) axis (Shen et al., 2021a). Mechanistically, *circ-NEIL3* regulates *ADAR1* expression by sponging *miR-432-5p* to induce RNA editing of glioma-associated oncogene 1 (*GLI1*), ultimately influencing cell cycle progression and promoting EMT in PDAC cells (Shen et al., 2021a). This process is regulated by the adenosine (A)-to-inosine (I) RNA editing *via* ADAR1 through a negative feedback loop (Solomon et al., 2017; Shen et al., 2021a). RNA editing is vital for preventing the abnormal activation of cytosolic nucleic acid-sensing pathways by self-double-stranded RNAs (Solomon et al., 2017). The editing effect on the RNA secondary structure is context-dependent, suggesting for the first time that circRNAs can interplay with RNA editing, underscored by the intricate regulatory role of ADAR1 on global RNA secondary structure (Solomon et al., 2017).

### 3.2 circ-0001666

*circ-0001666* (chr6:170,626,457-170,639,638) overexpression in PDAC increases transcription factor *SOX4* expression, a direct downstream effector of *miR-1251*, by binding to *miR-1251* (Zhang et al., 2021). Silencing *circ-0001666* repressed EMT in PDAC cells by upregulating *miR-1251* and downregulating *SOX4* (Zhang et al., 2021). This study indicated that *circ-0001666* functions *via* a ceRNA mechanism.

### 3.3 circ-0092367

*circ-0092367* (transcribed from the SNORD116-14 gene [ENSG00000206621]) is significantly downregulated in PDAC and inhibits EMT phenotypes and sensitizes PDAC cells to gemcitabine treatment both *in vitro* and *in vivo via* the *miR-1206*/epithelial splicing regulatory protein 1 (*ESRP1*) axis (Yu et al., 2021). ESRP1 regulates fibroblast growth factor receptor 2 (FGFR2)/K-sam-IIIb expression, an epithelial cell-specific FGFR2 isoform, and regulates hyaluronate receptor (CD44), catenin delta 1, and enabled homolog *Drosophila* splicing, which undergoes splicing changes during EMT (Vadlamudi et al., 2020). *circ-0092367* is involved in controlling EMT in PDAC and in the therapeutic response.

### 3.4 circ-0092314

*circ-0092314* (produced from human RAN binding protein 1 [RANBP1] gene located at chr22: 20,113,099-20,113,439) overexpression in PDAC induces EMT by sponging *miR-671*, increasing *S100P* expression (Shen et al., 2021b). *circ-0092314* directly binds to *miR-671* (Shen et al., 2021b). S100P may function as an ion sensor and contribute to cellular signaling by binding Ca^2+^, Zn^2+^, and Mg^2+^ (Suryavanshi et al., 2017). It interacts in a calcium-dependent manner with other proteins, such as villin 2, ezrin, and protein phosphatase 5 catalytic subunit, and indirectly plays a role in physiological processes, such as microvilli formation in epithelial cells (Suryavanshi et al., 2017).

### 3.5 circ-0005105

*circ-0005105* (produced at the yeast Sec24 homolog A [SEC24A] gene locus on chromosome 5q13 containing exon 9-12 [chr5:134022479-134023989]) overexpression in PDAC activates collagen type XI alpha 1 chain by targeting miR-20a-3p to promote PDAC progression (Ma et al., 2021). *circ-0005105* triggers EMT *via* a ceRNA mechanism, making it a potential prognostic marker and therapeutic target of PDAC (Ma et al., 2021). Future studies should investigate the mechanism by which this lncRNA influences EMT.

### 3.6 circ-UHRF1

*circ-UHRF1* (chr19:4,941,539-4,945,977) overexpression in PDAC regulates ADP ribosylation factor-like GTPase 4C expression by sponging *miR-1306-5p* to promote PDAC progression. *Circ-UHRF1* expression in PDAC cells was transcriptionally regulated *via* the interferon regulatory factor 3 (Liu et al., 2022).

## 4 Discussion

While clinical approaches indicated improvements in the first-line therapy, the 5-year overall survival only shows an increase from 5% to 10%, and surgical resection is only available for 20% patients with advanced PDAC, indicating that the disease is fatal for humans (Raja Arul et al., 2022). This cancer harbors the “big 4” driver mutations, i.e., substitutions or alterations in the KRAS proto-oncogene, GTPase (KRAS), TP53, cyclin*-*dependent kinase inhibitor 2A, and mothers against decapentaplegic homolog 4 (https://cancer.sanger.ac.uk/census; https://portal.gdc.cancer.gov). These can be useful for predicting the prognosis of patients with PDAC (McIntyre et al., 2020). Nevertheless, recent studies indicated that single-cell transcriptomes of PDAC exhibit several alterations; however, further studies are undoubtedly necessary for profiling whole-cell transcriptomes, including those of lncRNAs (Hwang et al., 2022). Altered lncRNA expressions can dysregulate clinically significant protein-coding of clinical significance and contribute to disease pathogenesis. lncRNA functions remain to be clearly understood. Some researchers have argued that lncRNAs are annotated in the wrong position and that they actually encode proteins, with several of them having been reported to encode peptides with biologically relevant functions, as demonstrated in the study involving *myoregulin*, an important regulator of skeletal muscle physiology (Anderson et al., 2015). The study revealed the possibility of additional micropeptides being encoded by the many RNAs that are currently annotated as noncoding (Anderson et al., 2015). For example, *LINC00961* encodes the small regulatory polypeptide of amino acid response (Matsumoto et al., 2017). Using the data in this review, we have significantly expanded our knowledge regarding lncRNAs, and we have a better understanding of their regulation of EMT in PDAC.

Notably, the current information indicates that almost all circRNAs are involved in the ceRNA mechanism, whereas linear lncRNAs are associated with diverse functions and not restricted to the ceRNA mechanism. The lncRNA–miRNA–mRNA ceRNA network has been implicated in various cancers, including lung (Wu et al., 2020), tongue (Zhou et al., 2019), and ovarian (Braga et al., 2020) cancers. The exact mechanism can be assessed by computational calculations and actual biological experiments showing the binding of miRNAs to circRNA *via* binding assays with mutated miRNA binding sites and luciferase translation readout as, similar to mRNAs, a significant portion of the noncoding transcriptome, including lncRNAs and pseudogenes, harbors miRNA-response elements that are targets of ceRNA (Karreth and Pandolfi, 2013). The fact that the lncRNA/circRNA–miRNA–mRNA ceRNA network can be predicted based on the overlap between miRNA-binding sites brings us one step closer to the complete functionalization of the human transcriptome in cancer, including PDAC, and toward precise diagnosis and innovative therapies for precision medicine. In combination with current development of PDAC surgery, we expected that the full characterization of lncRNA and circRNA network will contribute to further optimizing outcomes and shed light on refractory PDAC (Strobel et al., 2019).
